# Boosting Bupivacaine: The Role of Adjuvant Perineural Dexmedetomidine in Femoral Nerve Blocks

**DOI:** 10.7759/cureus.114092

**Published:** 2026-08-06

**Authors:** Ahila S, Gomathi Karmegam, Satish Raja M C, Ashwini G, Harsha Vardhanan T, Sahasyaa Adalarasan

**Affiliations:** 1 Emergency Medicine, Madras Medical College, Chennai, IND

**Keywords:** bupivacaine analgesia, emergency department analgesia, femoral nerve block, femur fracture analgesia, local anesthetic adjuvants, perineural dexmedetomidine, peripheral nerve block

## Abstract

Background

Effective pain management in patients with femoral fractures, such as intertrochanteric fractures, remains a major challenge in emergency medicine. Ultrasound-guided femoral nerve blocks administered on arrival have emerged as a promising alternative to conventional parenteral analgesics due to their ease of administration, rapid onset of analgesia, prolonged sensory blockade, and reduced risk of systemic adverse effects. Dexmedetomidine, a selective α2-adrenergic agonist, has been increasingly studied as an adjuvant to local anesthetics because of its ability to hasten the onset and prolong the duration of peripheral nerve blockade.

Aims and objectives

This study aimed to evaluate the efficacy of perineural dexmedetomidine as an adjunct to 0.25% bupivacaine in ultrasound-guided femoral nerve blocks for pain management in patients with femoral fractures presenting to the Department of Emergency Medicine, Rajiv Gandhi Government General Hospital.

Materials and methods

A single physician-blinded randomized controlled trial was conducted in the Department of Emergency Medicine, Rajiv Gandhi Government General Hospital. Thirty patients with femoral fractures were enrolled and randomly allocated into two groups comprising 15 patients each. Group B received an ultrasound-guided femoral nerve block using 19 mL of 0.25% bupivacaine with 1 mL of saline. In comparison, Group BD received 0.25% bupivacaine combined with 1 mL of perineural dexmedetomidine at a dose of 1 µg/kg. The onset of sensory blockade was assessed using the pinprick test, and pain intensity was evaluated using the Visual Analog Scale. Block duration was recorded separately. Hemodynamic parameters and adverse effects were also monitored. Statistical analysis was performed using SPSS Statistics version 26.0 (IBM Corp. Released 2019. IBM SPSS Statistics for Windows, Version 26.0. Armonk, NY).

Results

The addition of perineural dexmedetomidine to 0.25% bupivacaine resulted in a significantly faster onset of sensory blockade and prolonged duration of analgesia compared to bupivacaine alone. The mean onset of sensory blockade was 12 minutes, and the duration of analgesia was prolonged to 190 minutes in Group BD compared to 158 minutes in Group B (p = 0.001). Patients remained hemodynamically stable throughout the study period, and no significant adverse effects were noted.

Conclusions

The use of perineural dexmedetomidine as an adjunct to bupivacaine in ultrasound-guided femoral nerve blocks is associated with a faster onset and longer duration of analgesia in patients with femoral fractures presenting to the emergency department. This approach offers an effective and safe modality for acute pain management. Further studies with larger sample sizes are recommended to determine the optimal dose and maximize the therapeutic benefits of dexmedetomidine in peripheral nerve blocks.

## Introduction

Road traffic accidents are a major cause of emergency department admissions, causing femoral fractures such as intertrochanteric fractures. According to the trauma registry of the Department of Emergency Medicine, Rajiv Gandhi Government General Hospital, Chennai, road traffic accidents constituted nearly 40% of medicolegal cases and 9% of total emergency admissions during 2022, while self-fall injuries accounted for an additional 8% of cases. Isolated femoral fractures represented approximately 2% of all emergency department presentations during the same period. Globally, the burden of fractures has increased considerably over recent decades, with a reported 33% rise since 1990 and an estimated annual incidence of 188 million fractures worldwide in 2021 [1]. Femoral fractures constitute nearly 8% of all fractures and rank among the five most common fractures encountered in clinical practice. These fractures are broadly classified into femoral neck fractures [2], femoral shaft fractures [3], and distal femoral fractures [4].

In recent years, ultrasound-guided femoral nerve block has emerged as an effective and reliable modality for acute pain management in femoral fractures, alongside other regional anesthesia techniques, such as the sciatic nerve block [5]. The technique is relatively simple, provides rapid onset of analgesia, allows pain-free patient positioning and radiological assessment, and reduces the requirement for rescue analgesics [5-7]. However, systemic side effects with this method, such as bradycardia, hypotension, and sedation, should also be noted. Ultrasound guidance further improves the accuracy and safety of the procedure by enabling direct visualization of the femoral nerve and surrounding structures. Despite the efficacy of femoral nerve blocks, the duration of analgesia achieved with local anesthetic agents alone is often limited. Dexmedetomidine, a highly selective α2-adrenergic receptor agonist, has gained considerable interest as an adjuvant in regional anesthesia because of its sedative, anxiolytic, and analgesic properties [8,9]. Studies have demonstrated that dexmedetomidine, when used as an adjunct to local anesthetics in peripheral nerve blocks, shortens the onset of sensory blockade, prolongs the duration of analgesia, reduces opioid consumption, and improves patient comfort [9].

However, evidence regarding the use of perineural dexmedetomidine as an adjunct to bupivacaine in ultrasound-guided femoral nerve blocks for acute femoral fractures in emergency settings remains limited. Therefore, the present study was conducted to evaluate the efficacy and safety of dexmedetomidine as an adjunct to 0.25% bupivacaine in ultrasound-guided femoral nerve blocks in patients presenting with femoral fractures by evaluating variables such as onset time, Visual Analog Scale (VAS) score, pulse rate, respiratory rate, and mean arterial pressure (MAP).

## Materials and methods

This prospective single-blinded randomized controlled trial was conducted in the Department of Emergency Medicine, Rajiv Gandhi Government General Hospital, Chennai, over a one-year period from October 2023 to October 2024. Prior to the commencement of the study, approval was obtained from the Institutional Ethics Committee of Madras Medical College (approval number: 22092023). The study included adult patients aged 18 years or older who presented to the emergency department with isolated unilateral femoral fractures. Written informed consent was obtained from all eligible participants before enrolment into the study. Patients were excluded if they had a Glasgow Coma Scale (GCS) score of <12/15, polytrauma, associated head injury, nerve or vascular injury, coagulopathy, local site infection, compartment syndrome, hypovolemic shock, or were receiving anticoagulant therapy. All patients fulfilling the inclusion criteria during the one-year study period were recruited for participation.

A total of 30 eligible patients were enrolled and randomly allocated into two equal groups comprising 15 participants each using a computer-generated randomization sequence. Allocation concealment was ensured through sealed opaque envelopes, which were maintained by two independent researchers who were not involved in patient management. Participants assigned to Group BD received an ultrasound-guided femoral nerve block using 19 mL of 0.25% bupivacaine combined with dexmedetomidine at a dose of 1 µg/kg. Participants assigned to Group B received an ultrasound-guided femoral nerve block with 19 mL of 0.25% bupivacaine along with 1 mL of normal saline. The study was conducted using a single-blinded design, and blinding was maintained by preparing identical sterile syringes and using identical equipment for both study groups.

Following enrollment, all participants underwent a baseline assessment that included pain evaluation using the VAS. Routine physiological monitoring was also performed, including electrocardiography, pulse oximetry, respiratory rate, and non-invasive blood pressure measurements. Under strict aseptic precautions, an ultrasound-guided femoral nerve block was administered with the patient in the supine position. A high-frequency linear ultrasound probe was used to identify the femoral nerve, and the block was performed using an in-plane needle insertion technique.

The onset of sensory blockade was assessed using the pinprick test at one-minute intervals following administration of the femoral nerve block. Pain intensity during limb movement was evaluated using the VAS at 10-minute intervals. Hemodynamic parameters, including pulse rate, MAP, and respiratory rate, were monitored every five minutes during the first 30 minutes after block administration and subsequently every 30 minutes for a total duration of three hours. The primary outcome measure of the study was the onset of sensory blockade. Secondary outcome measures included the degree of pain relief, duration of analgesia, hemodynamic stability, and respiratory status, which were assessed by evaluating changes in VAS scores over time, pulse rate, MAP, and respiratory rate, respectively.

Statistical analysis was performed using SPSS Statistics version 26.0 (IBM Corp. Released 2019. IBM SPSS Statistics for Windows, Version 26.0. Armonk, NY). Continuous variables were summarized as mean ± standard deviation (SD) or median with interquartile range (IQR), depending on the data distribution, while categorical variables were presented as frequencies and percentages. The distribution of continuous variables was assessed using the Shapiro-Wilk test for normality. Friedman's repeated-measures analysis was used for repeated observations, and a p-value of <0.05 was considered statistically significant.

## Results

**Figure 1:**
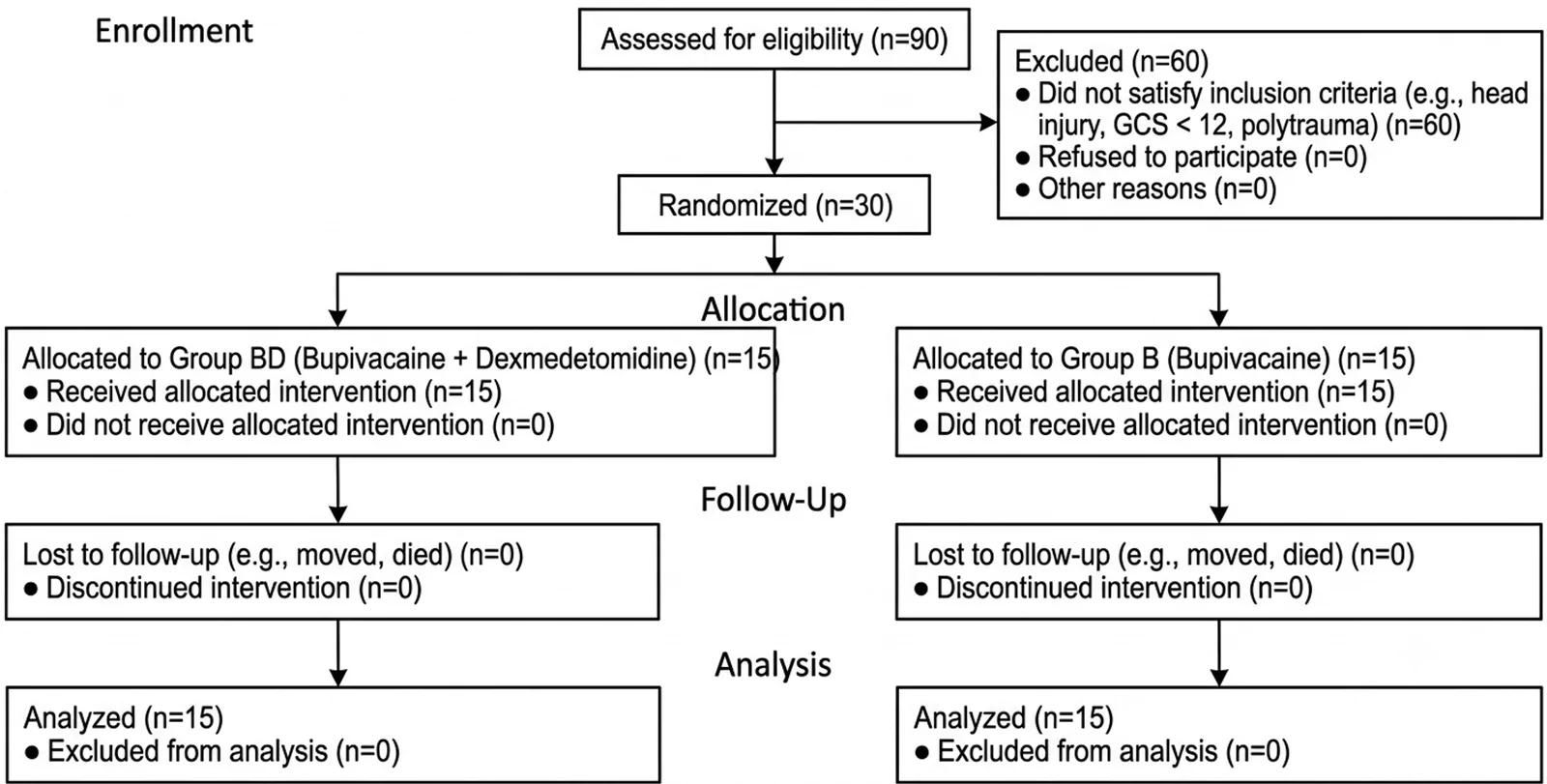
CONSORT flowchart This randomized controlled trial was reported in accordance with the Consolidated Standards of Reporting Trials (CONSORT) 2010 Statement [10].

A total of 30 patients with isolated unilateral femoral fractures were enrolled in the study and randomly allocated into two groups, with 15 patients in each group. The flow of participants through the trial is detailed in the CONSORT diagram below (Figure 1).

Descriptive statistics of the demographic variables in Group B and Group BD

The demographic characteristics of the study population are summarized in Table 1. A total of 30 patients with isolated unilateral femoral fractures were included in the study and were equally distributed into Group B and Group BD, with 15 patients in each group.

**Table 1:** Descriptive statistics of the demographic variables in Group B and Group BD Group B: patients who received bupivacaine, Group BD: patients who received bupivacaine with dexmedetomidine, N: number of patients in the group, RTA: road traffic accident

S. No.	Demographics	Group B, N = 15	Group BD, N = 15
1	Age (years)	55.13 ± 15.8	49 ± 24.6
2	Gender		
	Male	11 (57.9%)	8 (42.1%)
	Female	4 (36.4%)	7 (63.6%)
3	Height (cm)	164.67 ± 8.17	162.47 ± 6.5
4	Weight (kg)	74.27 ± 12.4	67.2 ± 10
5	Mode of injury		
	RTA	7 (43.8%)	9 (56.3%)
	Self-fall	8 (57.2%)	6 (42.9%)
6	Type of fracture		
	Neck of femoral fracture	3 (50%)	3 (50%)
	Intertrochanteric fracture	5 (55.6%)	4 (44.4%)
	Shaft of femoral fracture	3 (37.5%)	5 (62.5%)
	Distal femoral fracture	4 (57.1%)	3 (42.9%)

The demographic characteristics of both groups were comparable. The mean age was 55.13 ± 15.8 years in Group B and 49 ± 24.6 years in Group BD. Mean height and weight were slightly higher in Group B (164.67 ± 8.17 cm and 74.27 ± 12.4 kg) compared to Group BD (162.47 ± 6.5 cm and 67.2 ± 10 kg). Overall, the demographic profile and injury characteristics were similar between the two groups. Intertrochanteric fractures were the most common in Group B, whereas shaft femoral fractures predominated in Group BD. Neck of femur and distal femoral fractures were distributed similarly between the groups. The overall fracture pattern remained comparable between both study groups.

Comorbidities in Group B and Group BD

The distribution of comorbidities was comparable between the two groups. The common comorbidities observed were type 2 diabetes mellitus (T2DM), systemic hypertension (SHTN), and coronary artery disease (CAD). In Group B, T2DM, SHTN, and CAD were present in 26%, 33%, and 33% of patients, respectively, whereas in Group BD, they were observed in 20%, 20%, and 13% of patients, respectively. A greater proportion of patients had no associated comorbidities in Group BD (53%) compared to Group B (40%). Overall, 14 patients had no comorbid illnesses. This is shown in Table 2.

**Table 2:** Frequency and percentage distribution of comorbidities in Group B and Group BD Group B: patients who received bupivacaine, Group BD: patients who received bupivacaine with dexmedetomidine, N: number of patients, T2DM: type 2 diabetes mellitus, SHTN: systemic hypertension, CAD: coronary artery disease

S. No.	Comorbidities	Group B, N = 15	Group BD, N = 15
		Frequency (%)	Frequency (%)
1	T2DM	4 (26%)	3 (20%)
2	SHTN	5 (33%)	3 (20%)
3	CAD	5 (33%)	2 (13%)
4	No comorbidities	6 (40%)	8 (53%)

Clinical parameters observed in Group B and Group BD

Onset and Duration of the Femoral Nerve Block in Both Groups

Group BD demonstrated a significantly faster onset and a significantly longer duration of sensory blockade than Group B. The mean onset of sensory blockade was faster in Group BD (12.27 ± 2.8 minutes) compared to Group B (21.6 ± 4.2 minutes). Similarly, the duration of sensory blockade was prolonged in Group BD (190.4 ± 21.2 minutes) when compared with Group B (158 ± 23.4 minutes). These differences were statistically significant (p = 0.001), suggesting that dexmedetomidine, when used as an adjuvant, improves both the onset and duration of femoral nerve block. This is shown in Table 3.

**Table 3:** Comparison of sensory blockade onset and duration in Group B and Group BD * Statistically significant (p < 0.05), ^a ^Mann-Whitney U test, Student's t-test Group B: patients who received bupivacaine, Group BD: patients who received bupivacaine with dexmedetomidine, SD: standard deviation, IQR: interquartile range, CI: confidence interval

S. No.	Parameter	Mean ± SD		p-value
		Group B, N = 15 (minutes)	Group BD, N = 15 (minutes)	
1	Onset of sensory blockade^a^	21.6 ± 4.2	12.27 ± 2.8	0.001*
2	Duration of sensory blockade	158 ± 23.4	190.4 ± 21.2	0.001*

VAS Score After the Femoral Nerve Block in Both Groups

**Figure 2:**
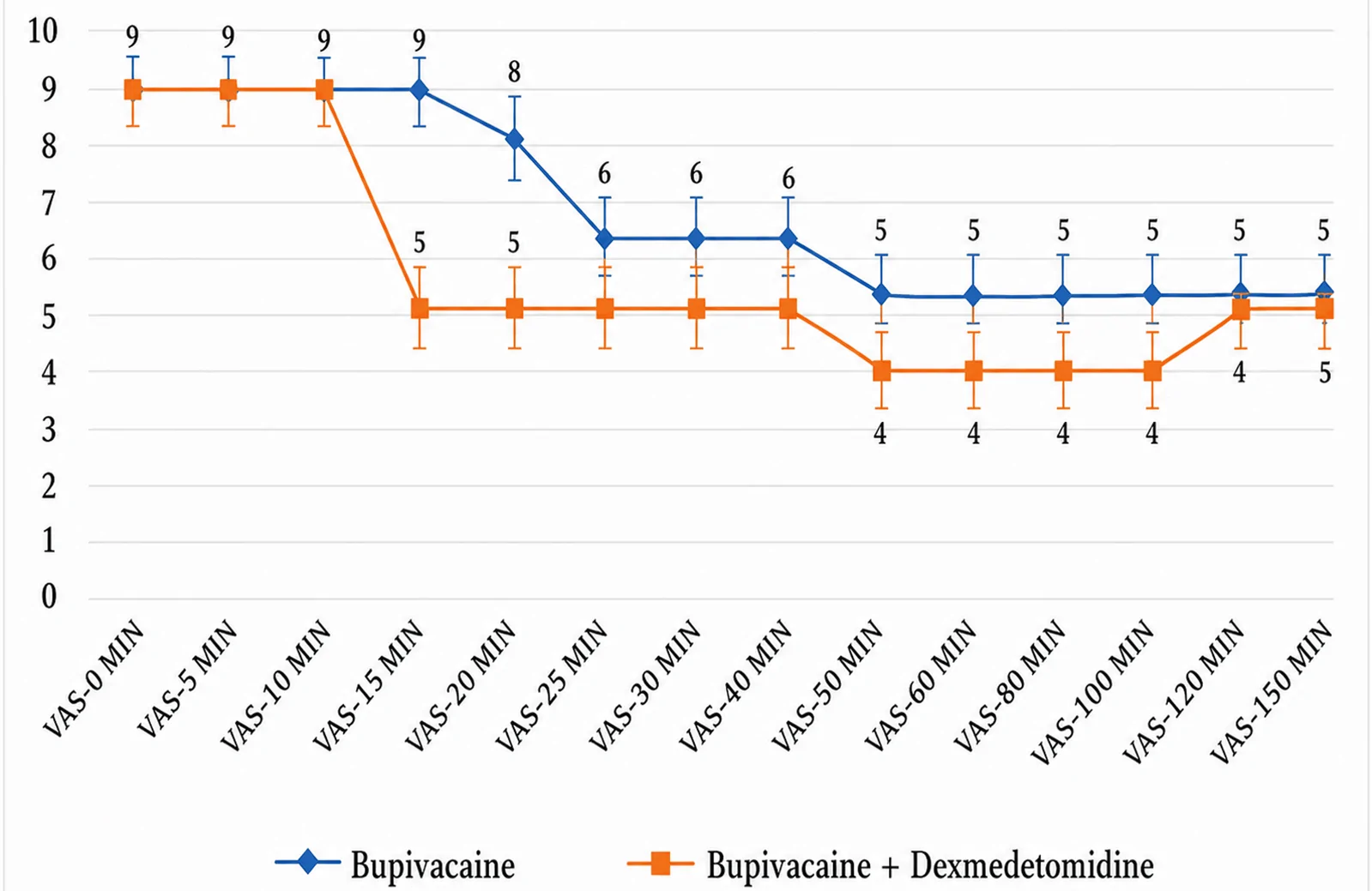
Mean VAS score in Group B and Group BD at rest Group B: patients who received bupivacaine, Group BD: patients who received bupivacaine with dexmedetomidine, VAS: Visual Analog Scale

In Group B, Friedman's repeated measures analysis showed a significant reduction in pain scores over time (p = 0.001). VAS scores remained high during the initial 15 minutes and gradually declined from 20 minutes onward, reaching a median score of 6 at 25-30 minutes and stabilizing at 5 from 50 minutes onward. Post-hoc pairwise comparisons with Bonferroni correction showed significant differences between the early and later time points (adjusted p < 0.05), indicating effective analgesia beginning around 25-30 minutes. This is shown in Figure 2.

In contrast, Group BD demonstrated an earlier and greater reduction in VAS scores, with meaningful pain relief observed within 10-15 minutes. Pain scores remained consistently lower throughout the observation period compared to Group B, suggesting that dexmedetomidine accelerated the onset and prolonged the duration of analgesia. These findings support dexmedetomidine as an effective adjuvant to bupivacaine for improving postoperative analgesia and patient comfort in femoral nerve block.

MAP After the Femoral Nerve Block in Both Groups

**Figure 3:**
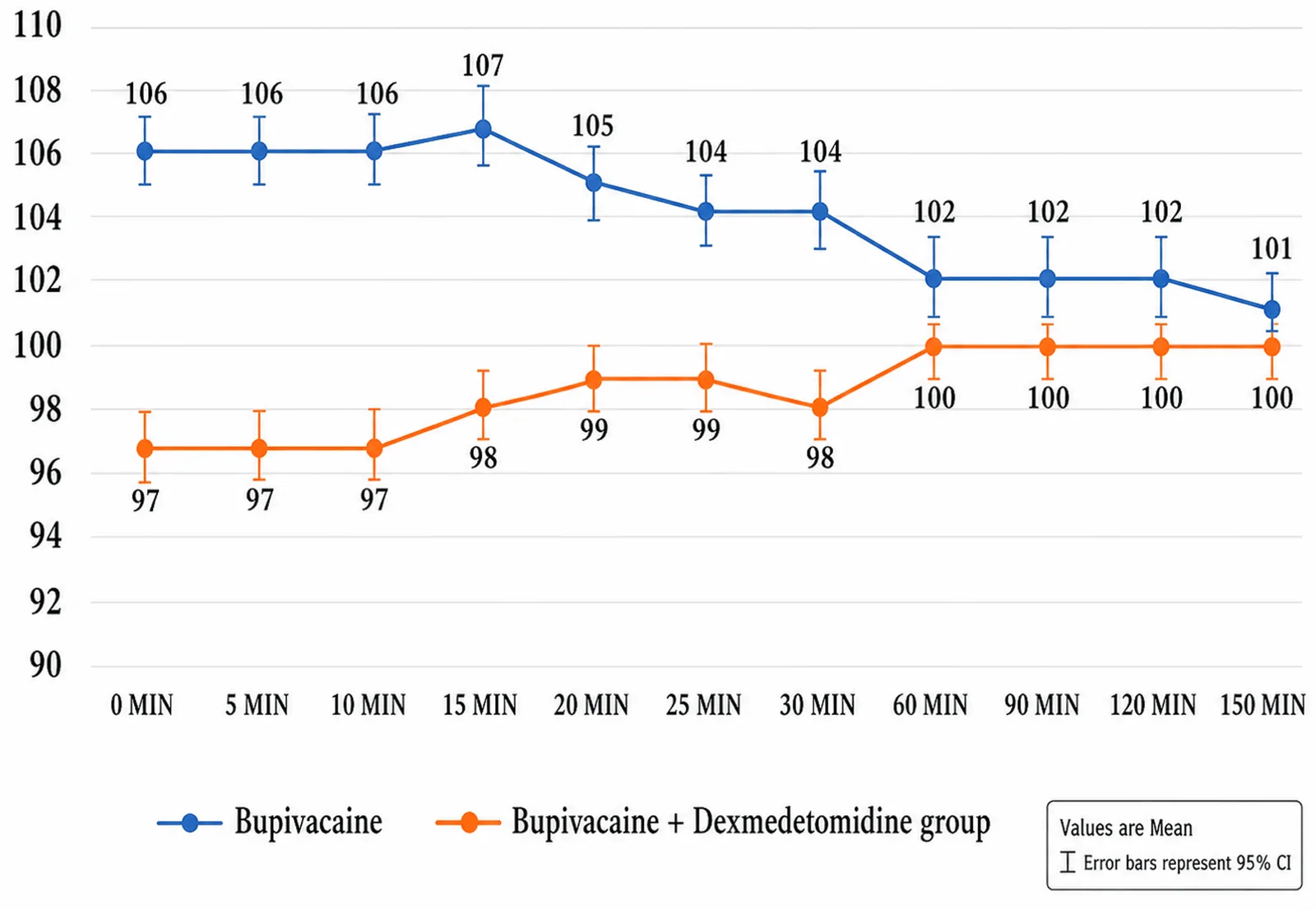
MAP at different points in Group B and Group BD Group B: patients who received bupivacaine, Group BD: patients who received bupivacaine with dexmedetomidine, MAP: mean arterial pressure, CI: confidence interval

MAP remained largely stable in both groups throughout the observation period following femoral nerve block. In Group B, MAP showed a gradual declining trend over time, decreasing from a baseline value of 106.13 ± 13.55 mmHg to 100.89 ± 12.63 mmHg at 150 minutes. Although a mild reduction was observed after 15-20 minutes, pairwise comparison analysis did not demonstrate statistically significant differences between individual time intervals after Bonferroni correction, indicating overall hemodynamic stability despite the temporal trend. This is shown in Figure 3.

In Group BD, MAP values remained comparatively more stable, ranging from 96.93 ± 11.73 mmHg at baseline to 100.27 ± 7.01 mmHg at 150 minutes. A slight increase in MAP was observed during the early period following block administration, after which values remained steady with reduced interindividual variability. Friedman’s two-way ANOVA showed no statistically significant variation in MAP over time (p = 0.556). These findings suggest that adding dexmedetomidine to bupivacaine provides effective analgesia without causing clinically significant hemodynamic fluctuations, thereby maintaining stable peri-procedural blood pressure control.

Pulse Rate After the Femoral Nerve Block in Both Groups

**Figure 4:**
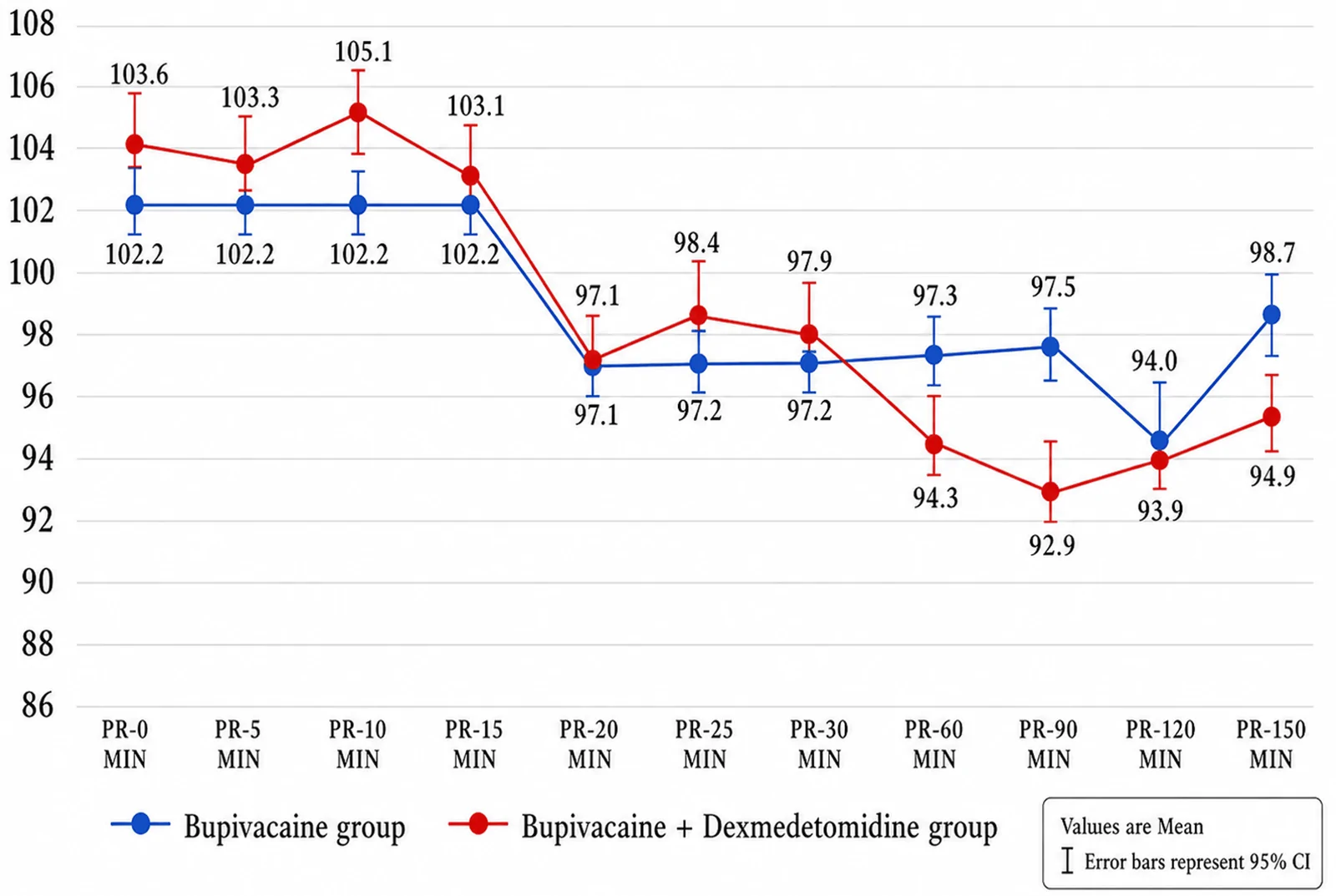
Pulse rate at different points in Group B and Group BD Group B: patients who received bupivacaine, Group BD: patients who received bupivacaine with dexmedetomidine, CI: confidence interval

In Group B, the mean pulse rate remained around 102 bpm during the initial 15 minutes and gradually declined thereafter, reaching 96 bpm at 120 minutes before slightly increasing at 150 minutes. Repeated measures analysis showed a significant temporal change in pulse rate, with the decline becoming more evident after 20 minutes, although pairwise comparisons between individual intervals were not statistically significant. Overall, the pulse rate remained hemodynamically stable throughout the observation period. This is shown in Figure 4.

In Group BD, a similar trend was observed, with an initial mild rise in pulse rate at 10 minutes (105.07 bpm) followed by a gradual decline from 20 minutes onward, reaching the lowest mean value at 90 minutes (92.93 bpm). The reduction in pulse rate was more pronounced in the dexmedetomidine group, likely reflecting the sympatholytic action of dexmedetomidine. However, no clinically significant bradycardia or hemodynamic instability was observed. These findings suggest that the addition of dexmedetomidine to bupivacaine provides effective analgesia while maintaining acceptable cardiovascular stability.

Respiratory Rate After the Femoral Nerve Block in Both Groups

**Figure 5:**
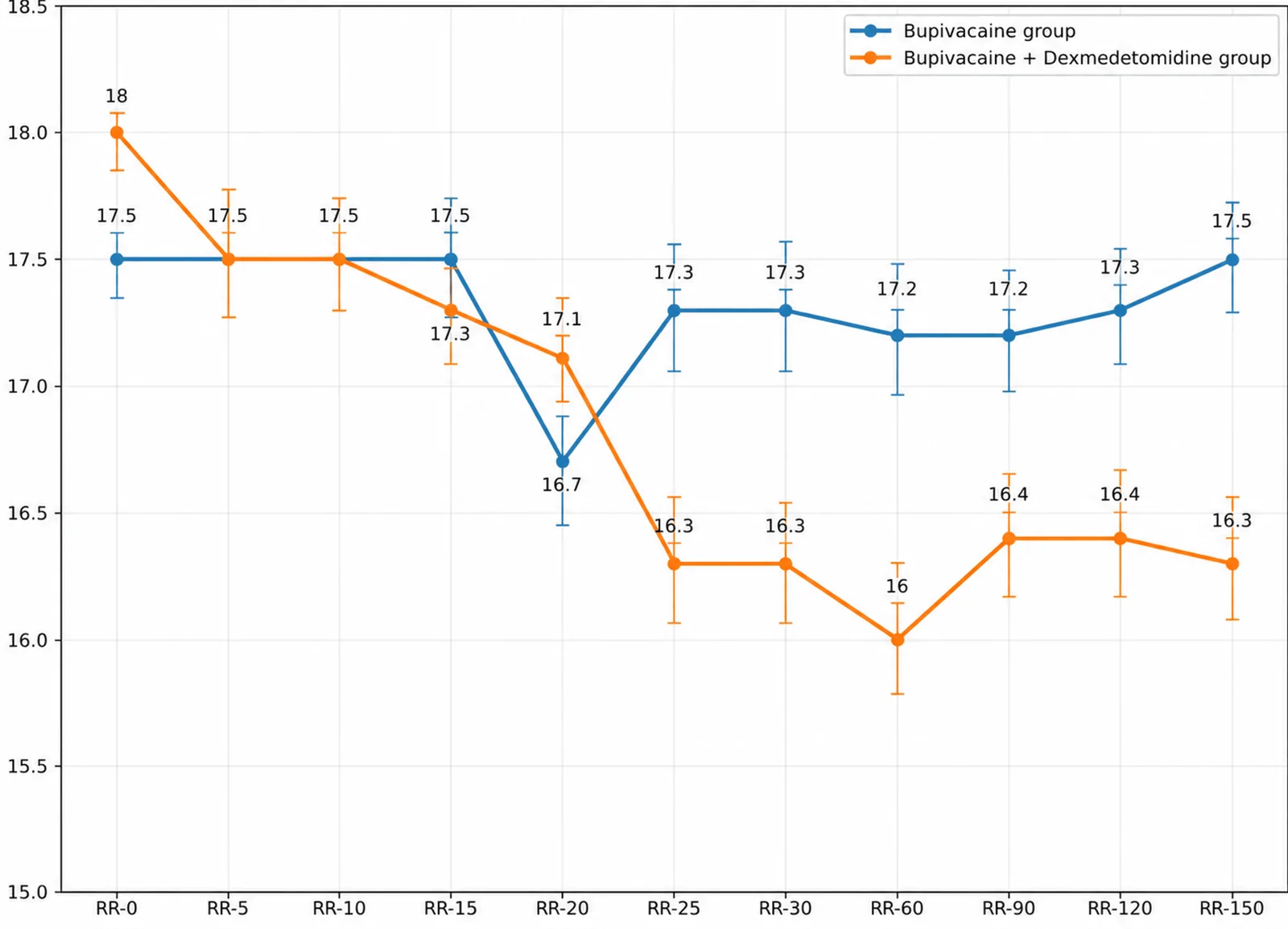
Respiratory rate at different points in Group B and Group BD Group B: patients who received bupivacaine, Group BD: patients who received bupivacaine with dexmedetomidine

In Group B, the respiratory rate remained relatively stable throughout the observation period, with mean values ranging between 16 and 17 breaths/minute. A mild transient decline was observed at 20 minutes, following which the respiratory rate returned to near-baseline values and remained stable up to 150 minutes. Friedman’s repeated measures analysis demonstrated a statistically significant variation in respiratory rate over time (p < 0.05). However, post-hoc pairwise comparisons did not reveal statistically significant differences between individual time intervals, suggesting that the observed changes were minimal and not clinically significant. This is shown in Figure 5.

A similar trend was observed in Group BD, where respiratory rates remained within normal physiological limits throughout the study period. Despite the addition of dexmedetomidine, no clinically significant respiratory depression or oxygen desaturation was observed. Friedman analysis in the dexmedetomidine group also showed temporal variation in respiratory rate, although the overall changes were small and clinically stable. These findings indicate that dexmedetomidine as an adjuvant to bupivacaine in ultrasound-guided femoral nerve block provides effective analgesia without significant respiratory compromise, supporting its safety and hemodynamic tolerability in patients with femoral fractures.

## Discussion

Femoral fractures are among the most painful orthopedic injuries encountered in the emergency setting and are associated with considerable morbidity, especially when pain is not addressed early. Early pain control is often poor because of concerns regarding opioid adverse effects, communication difficulties in elderly patients, and inconsistent administration of prescribed analgesics [11,12]. Regional techniques such as femoral nerve block are particularly valuable because they provide rapid and targeted analgesia without the sedative and respiratory depressant effects seen with systemic opioids [12,13].

Femoral nerve block is increasingly used as an on-arrival analgesic technique in femoral fractures because it is simple, rapid, and provides effective site-specific pain relief without significant sedation or respiratory depression [14]. Early pain management is essential, as inadequately treated pain can increase morbidity, delay mobilization, and worsen patient outcomes [11]. Conventional analgesics used in femoral fractures include paracetamol, NSAIDs, and opioids such as morphine and fentanyl. Still, opioid use is often limited by adverse effects like nausea, delirium, sedation, and respiratory depression, especially in elderly patients [15]. Compared to systemic analgesia, femoral nerve block provides better pain control, reduces muscle spasm and opioid requirement, and improves patient comfort during positioning and perioperative care. Various adjuvants such as clonidine, dexmedetomidine, and adrenaline may also be added to local anesthetics to enhance the quality and duration of the block [13,15]. The injury activates peripheral nociceptors, particularly A-delta and C fibers, leading to the release of inflammatory mediators such as prostaglandins, bradykinin, endothelin, cytokines, and nerve growth factor (NGF) at the fracture site. NGF binds to tropomyosin receptor kinase A (TrkA) receptors on sensory neurons, and the resulting NGF-TrkA complex travels retrogradely to the spinal cord, where pain signals are transmitted through the anterolateral pathway and perceived as pain [16].

Dexmedetomidine is a highly selective α2-adrenoceptor agonist with greater affinity for α2 receptors than clonidine [8]. Clinically, it provides sedation, anxiolysis, and opioid-sparing analgesia without significant respiratory depression [8]. Common side effects experienced with dexmedetomidine include hypotension, bradycardia, and giddiness [17]. Owing to these properties, dexmedetomidine is commonly used as an adjuvant with local anesthetics such as bupivacaine and ropivacaine to improve the onset, quality, and duration of peripheral nerve blocks [18-20].

Comparison with other similar studies

Previous studies have consistently demonstrated that dexmedetomidine, when used as an adjuvant with local anesthetics, improves the quality of peripheral nerve blocks by producing faster onset and prolonged duration of analgesia. Agarwal et al. reported that the addition of dexmedetomidine to bupivacaine resulted in earlier onset and prolonged analgesic duration compared to bupivacaine alone, findings that are consistent with the present study [21]. Similar observations were reported by Tripathi et al., where dexmedetomidine provided earlier sensory blockade and longer duration of action compared to clonidine in supraclavicular brachial plexus block [22].

The findings of the present study are also supported by dose-related studies on dexmedetomidine. Abdulatif et al. demonstrated that increasing doses of dexmedetomidine improved the onset and duration of sensory blockade, although higher doses were associated with greater adverse effects [23]. In the present study, a statistically significant reduction in VAS scores was observed within 20 minutes in the dexmedetomidine group, indicating a rapid onset of analgesia and sustained pain relief. Similar findings were reported by Helal et al., who observed reductions in heart rate and blood pressure, likely due to α2-mediated sympatholytic effects and improved pain control [24]. Overall, the present study supports the growing evidence that dexmedetomidine is an effective and safe adjuvant for femoral nerve block, providing prolonged analgesia with a stable hemodynamic profile [21-24].

Limitations

The present study has certain limitations. The sample size of the study was relatively small, with only thirty patients included, which may limit the generalizability of the findings to the larger population. Additionally, the short follow-up period, baseline imbalances, and the heterogeneous fracture types and varying injury severity represent important limitations. The study was conducted in a single tertiary care center over a limited duration; therefore, the results may not represent outcomes in different healthcare settings. Since the study included only isolated unilateral femoral fractures, the effectiveness of dexmedetomidine in polytrauma patients or patients with associated systemic injuries could not be evaluated. Long-term follow-up regarding postoperative analgesic requirements, functional recovery, and delayed adverse effects was not assessed. Additionally, only a single dose of dexmedetomidine was studied; therefore, the ideal dose and dose-response relationship could not be determined.

This study was also limited by the method used to assess sensory blockade. Pinprick testing was used for evaluating the onset and duration of sensory blockade, which is subjective and dependent on patient response. More objective methods, such as a nerve stimulator, could provide better accuracy in confirming the onset and duration of sensory blockade.

## Conclusions

The present study demonstrates that perineural dexmedetomidine at 1 mcg/kg, as an adjunct to 0.25% bupivacaine in an ultrasound-guided femoral nerve block, significantly improves the quality of analgesia in patients presenting with femoral fractures. The addition of dexmedetomidine resulted in a faster onset of sensory blockade and a prolonged duration of analgesia when compared to bupivacaine alone. The average duration of effective analgesia in the dexmedetomidine group was approximately 180 minutes, thereby providing sustained pain relief during the acute management phase in the emergency department.

Patients who received dexmedetomidine showed appreciable pain relief within 20 minutes following administration of the nerve block. In addition to superior analgesic efficacy, the combination maintained stable hemodynamic and respiratory parameters throughout the study period without significant adverse effects such as bradycardia, hypotension, excessive sedation, or respiratory compromise. These findings indicate that dexmedetomidine is a safe and effective adjuvant for femoral nerve block in patients with acute femoral fracture.

This study, conducted in the Department of Emergency Medicine, highlights the importance of early ultrasound-guided femoral nerve block as part of multimodal pain management in trauma care. Adding dexmedetomidine to bupivacaine not only improves patient comfort and facilitates easier examination and limb manipulation but also contributes to improved overall patient care in the emergency setting. Further studies with larger sample sizes are recommended to determine the optimal dose and maximize the clinical benefits of dexmedetomidine in peripheral nerve blocks.
